# Revealing the impact of built environment, air pollution and housing price on health inequality: an empirical analysis of Nanjing, China

**DOI:** 10.3389/fpubh.2023.1153021

**Published:** 2023-08-17

**Authors:** Yu Ding, Chenglong Wang, Jiaming Wang, Peng Wang, Lei Huang

**Affiliations:** ^1^Faculty of Civil Engineering and Mechanics, Jiangsu University, Zhenjiang, China; ^2^State Key Laboratory of Pollution Control and Resource Reuse, School of the Environment, Nanjing University, Nanjing, China

**Keywords:** built environment, health inequalities, air pollution, residential segregation, housing price

## Abstract

**Introduction:**

Residential segregation have become a common phenomenon in China recently. Socioeconomically disadvantaged residents were more likely to live in communities with higher PM_2.5_ concentrations and poorer built environment, which may ultimately lead to a higher health risk, further exacerbating health inequalities. However, the reasons for health inequalities under residential segregation remain unclear.

**Methods:**

This study quantified the built environment, air pollution, mortality rate and housing price at 1 km × 1 km grid scale. Moderating effect model, mediating effect model, moderated mediating effect model were used to progressively clarify the relationship between the four.

**Results:**

Results show that, in terms of spatial distribution, the central area has high housing price with good built environment, low PM_2.5_ concentration and low mortality rate. While the suburban area has low housing price, poor built environment, high PM_2.5_ concentration and high mortality rate. Additionally, built environment can not only reduce health risks through moderating effect, but also affect health through the mediating effect of PM_2.5_. There is heterogeneity in moderating effect of built environment in different locations. Housing prices can moderate the effect of built environment on health. This study would offer important reference for urban planning to mitigate the effect of built environment inequalities on health inequalities in China.

## 1. Introduction

Air pollution poses a significant threat to public health. Ninety percent of the world’s population resides in areas where air quality exceeds World Health Organization (WHO) standards (1). PM_2.5_ is a key pollutant among numerous hazy air pollutants that negatively affect human health, as it can deposit hazardous substances such as sulfates and heavy metals in the airways and respiratory tracts of humans (2). Every year, millions of people die prematurely due to exposure to ambient PM_2.5_. In China, a study based on monitored concentrations and an integrated exposure response model estimated that 740,140 [95% confidence interval (CI): 646,538–839,968] premature fatalities in 2020were attributable to PM_2.5_ (3). Therefore, mitigating the impact of air pollution on human health has become a critical public health issue (4).

Built environment, which is defined as human-made physical environment surroundings and conditions, has been deemed a decisive factor in the health of city dwellers (5). Recent studies have demonstrated that built environments can mitigate the adverse health effects of air pollution. It has proved that built environment in Nanjing moderated the relationship between air pollution and non-accident mortality (6). Specifically, SHDI and water area can mitigate the effect of PM_2.5_ on all-cause mortality (6). Additionally, it has been estimated that the aggregate impact of vegetation on PM_2.5_ in the UK is about 1% (7). The quantity and spatial allocation of green space play a crucial role in urban PM_2.5_ management (8). More than that, it has been confirmed that the built environment can directly affect the concentration of air pollutants in urban areas. For example, high density buildings may hinder the dispersion of air pollutants, resulting in the accumulation of pollutants (9). Increased impervious surface increases temperatures, which may contribute to the formation of air pollutants (10).

In addition, studies that accounted for built environments, air pollution, and health risks revealed intriguing spatial differences. Areas with high housing prices are mainly concentrated around major urban green space can effectively reduce particulate pollution, realize the ecological benefits of high-density urban areas, and improve human health in areas where housing price is high (8). In contrast, industrial toxic pollutants are frequently concentrated and accumulated in low-income communities with less expensive housing, further increasing the health burden in these communities, which already suffer from multiple deprivations (2). Notably, housing prices may also play a role in the relationship between the three, and may be one of the variables that can explain disparities in health in the context of residential segregation.

Health, built environment, air pollution, and housing prices are interdependent and cannot be separated. To mitigate the health risk of urban residents, it is urgent to understand the mechanism of influence of the four factors. However, current researches on built environment and health focus primarily on identifying influencing factors, exploring influencing pathways, and comparing influencing effects. Majority of studies only considered the relationship between indicators of built environment and air pollution or built environment and specific diseases. Few studies consider the relationship between the three in depth. Lack of research examines the characteristics of built environment and its environs, such as the location distribution, pollutant concentration, and economic value.

To fill that gap, this study began by examining the relationship among built environment, PM_2.5_ and mortality rate in order to progressively clarify the relationship between the four in various areas. Then, taking into account housing price, relationship between the four was investigated further. The subsequent efforts were made: the study examined (i) the moderating effect of built environment on the relationship between air pollution and mortality rate (ii) the mediating effect of air pollution between built environment and mortality rate, and (iii) the moderating effect of housing price on the air pollution-mediated relationship between built environment and mortality rate.

## 2. Materials and methods

### 2.1. Study area

The capital of Jiangsu province in China, Nanjing (31°14′ N–32°37′ N, 118°22′ E–119°14′ E), is one of the main regional central cities with a high population density and well-developed industry (11). The majority of the petrochemical businesses in Nanjing are located within the city. There are many petrochemical enterprises in Nanjing, mainly distributed around the urban area., Additionally, the enclosed terrain encircled by mountains on three sides and water on one side contributes to the accumulation of air pollutants in the urban area. PM_2.5_ has been identified as a key factor in reducing air pollution in the Yangtze River Delta, where Nanjing is situated (12).

**Figure 1:**
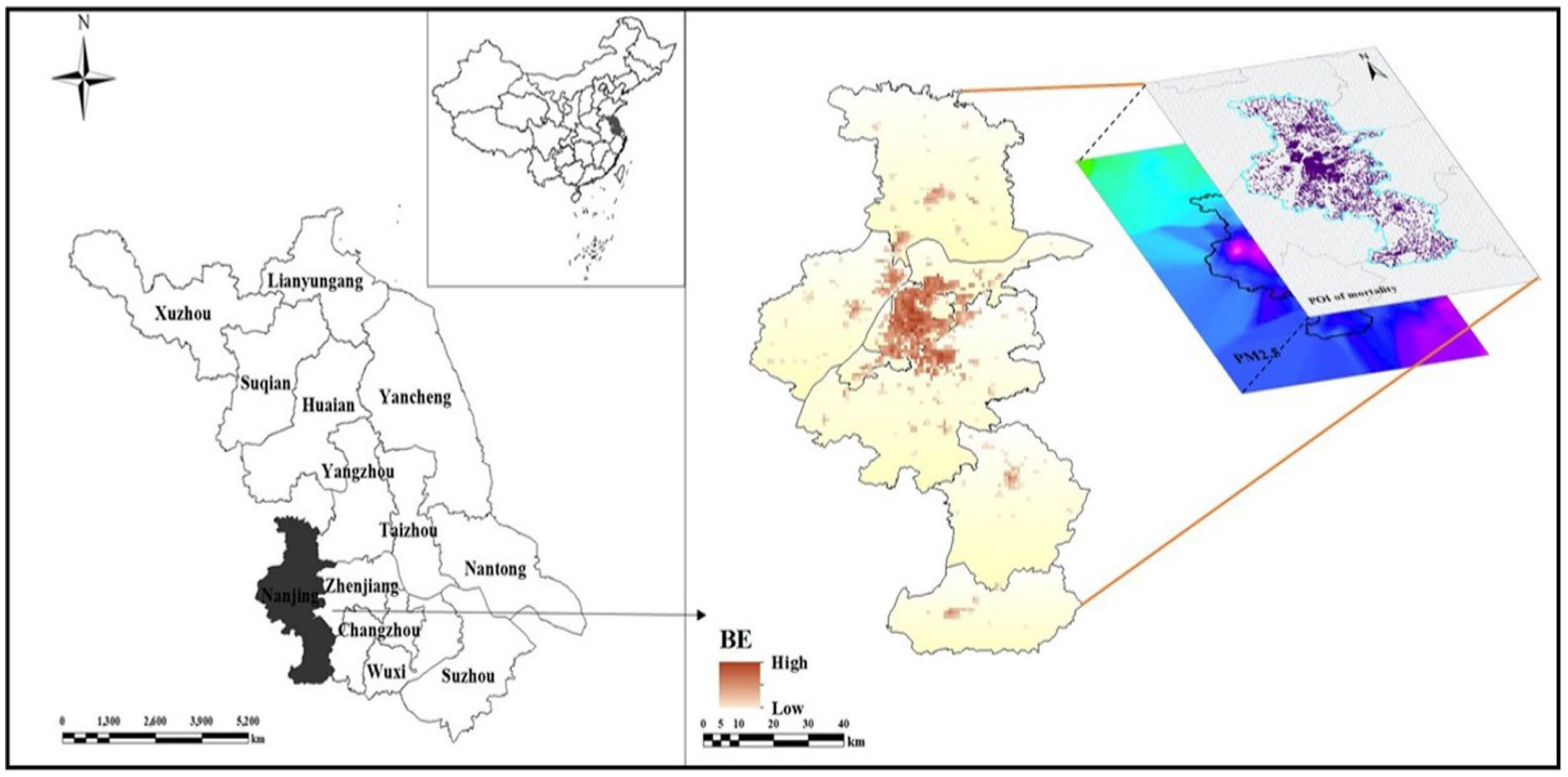
Location of the study area.

Nanjing is the “ancient capital of six dynasties.” The history of urban development over a millennium has had a profoundly impact on the spatial differentiation pattern of Nanjing. South of the city center, the majority od, residential areas and subdivisions are situated along the Qinhuai River. Through the spatial pattern of urban functions was consistently reinforced as a result of the construction of capital cities during multiple dynasties (13). This century, residents of working class remained concentrated in the southern part of the inner city (14). Consequently, education, health care, commerce and other public services are centralized in the city center. Nanjing consists of five urban areas (Qinhuai, Xuanwu, Gulou, Jianye, Yuhuatai), five suburban areas (Qixia, Pukou, Jiangning, Gaochun, Liuhe). As the overall study area, Nanjing City was divided into 1 km × 1 km grids for the investigation (Figure 1).

### 2.2. Data collection

**Figure 2:**
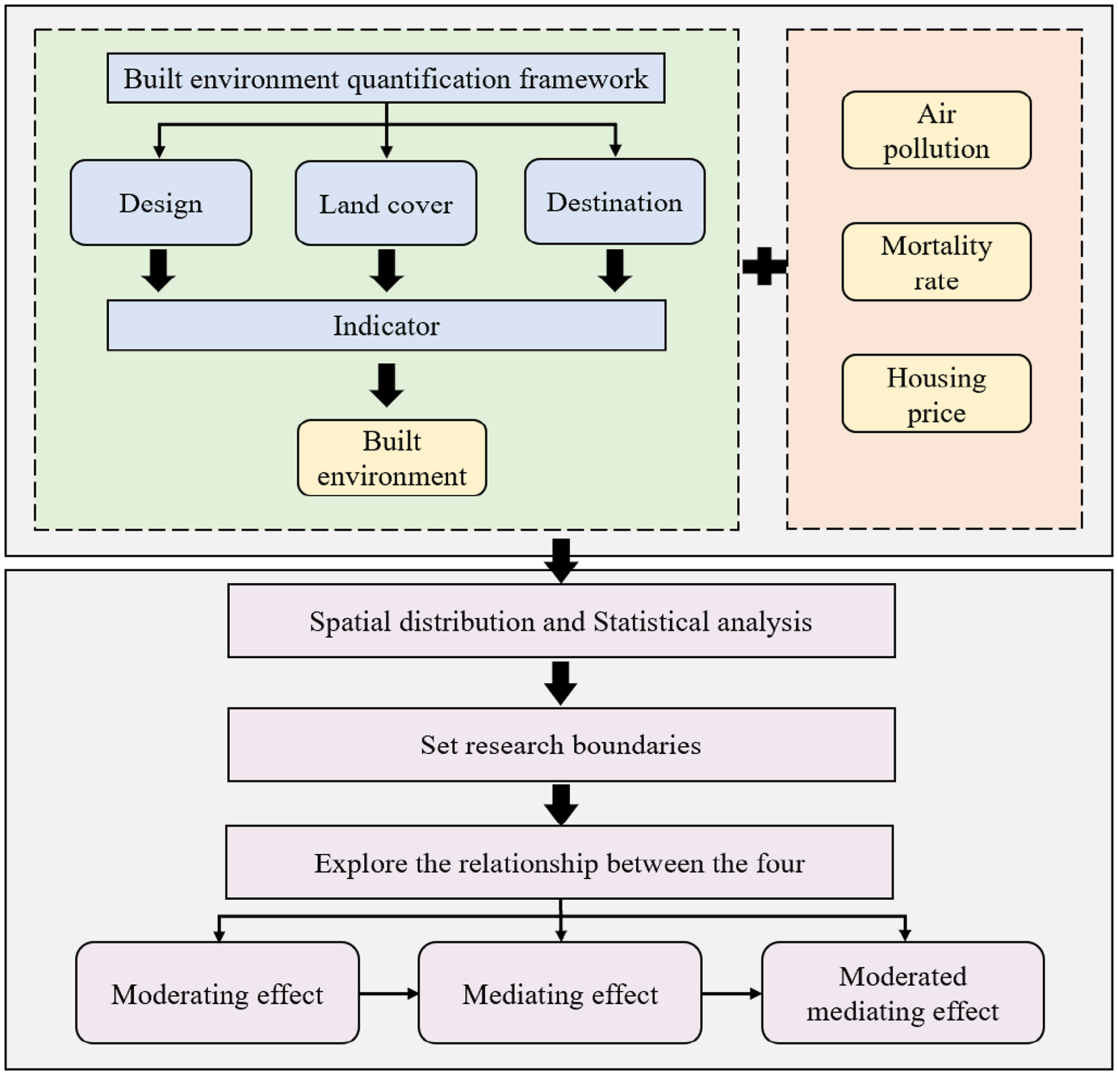
Framework of the study.

In this study, we utilized road data, building information, the Normalized Difference Vegetation Index (NDVI), point of interest (POI), land use and land cover data, air pollution (PM_2.5_), population density, mortality data and the 2020 housing price. Road data from OpenStreetMap road data was used to compute road crossing and road density at a grid scale. The building dataset was got from BIGEMAP map platform,^1^ which included information on footprints, building space coordinates and the number of stories in a structure. Point of interest (POI) data from BIGEMAP was acquired in order to assess the diversity of POI and the quantity of grid facilities. NDVI was used to measure surface greenness in this study. Based on the land cover data from Resource and Environment Science and Date Center, the study computed the proportion of water body, construction land and forestland within the grid. Figure 2 depicts the framework of the study.

#### 2.2.1. Built environment

This study developed a framework for assessing the built environment (Table 1) including design, destination, and land cover dimensions (15). Shannon’s Diversity Index (SHDI) was used to quantify the degree of land use diversity. Hill Numbers equation was used to calculate the diversity of POI (16).


$$D={\left(\overset{n}{\underset{i=1}{\sum }}P_{i}^{q}\right)}^{1/\left(1-q\right)}$$

where D is diversity, n indicates the number of the types, $P_{i}$ is proportion of the category occupied by type i, and *q* = 2 (according to Gini-Simpson Concentration index).

**Table 1:** Description of 15 indexes of the built environment.

Category	Index	Description of index
Design	Building density (BD)	The ratio of the building footprint area to grid area
	NDVI	The greenness within the grid
	Road density (RD)	The ratio of roads within the grid
	Road crossing (RC)	Number of road crossings within the grid
Destination	Hygiene facility (HF)	Number of hospital and health care facilities within the grid
	Industrial Park (IP)	Number of Industrial parks within the grid
	Sports facility (SF)	Number of gym and fitness room within the grid
	Traffic facility (TF)	Number of bus station and subway station within the grid
	Gas station (GS)	Number of gas stations within the grid
	Diversity of POI (DPOI)	The POI mix
Land cover	Construction land (CL)	Proportion of construction land within the grid
	Forestland (FL)	Proportion of forestland within the grid
	Water area (WA)	Proportion of water area within the grid
	Shannon’s diversity index (SHDI)	Diversity of land cover

#### 2.2.2. Air pollution

According to high-resolution spatiotemporal model and data from previous researches (14, 17), this study calculated the annual PM_2.5_ at 1 km × 1 km grid scale in Nanjing.

#### 2.2.3. Residential data

Housing prices of the residential communities were obtained from Lian Jia,^2^ an online housing platform that provides real estate and rental services in China. Then, the BIGEMAP was used to calibrate the obtained location information from the residential community.

#### 2.2.4. Mortality

This study collects the mortality data of non-accidental causes (A00-R99) in 2020 from the Jiangsu Provincial Center for Disease Prevention and Control (18, 19), which is classified by the International Statistical Classification of Disease and Related Health Problems, 10th Revision.^3^

Compared to using medical data such as morbidity and hospital admissions (20, 21) to depict the average health outcome of an area, the POI data of mortality used in this study can more accurately characterize the personal health status at 1 km × 1 km grid scale. Previous studies have used grid mortality data (22, 23) to represent health outcomes and examine the health burden due to ambient pollution.

### 2.3. Statistical analysis

Before statistical analysis, the sample with a negative mortality rate and unreachable housing price was eliminated from this study. Secondly, correlation analysis is used to explore the relationships between the air pollution, built environment, housing price and mortality rate. Thirdly, based on the correlation test, regression models were used to determine positive and negative indexes by examining the relationships between built environment indicators and mortality rate. The correlation between two variables, x and y, can be measured using Pearson’s correlation coefficient. The calculation for the coefficient r is as follows:


$$\begin{array}{c} r=\frac{\sum_{i=1}^{n}\left(x_{i}-\bar{x}\right)\left(y_{i}-\bar{y}\right)}{\sqrt{\sum_{i=1}^{n}{\left(x_{i}-\bar{x}\right)}^{2}\sum_{i=1}^{n}{\left(y_{i}-\bar{y}\right)}^{2}}} \end{array}$$

**Figure 3:**
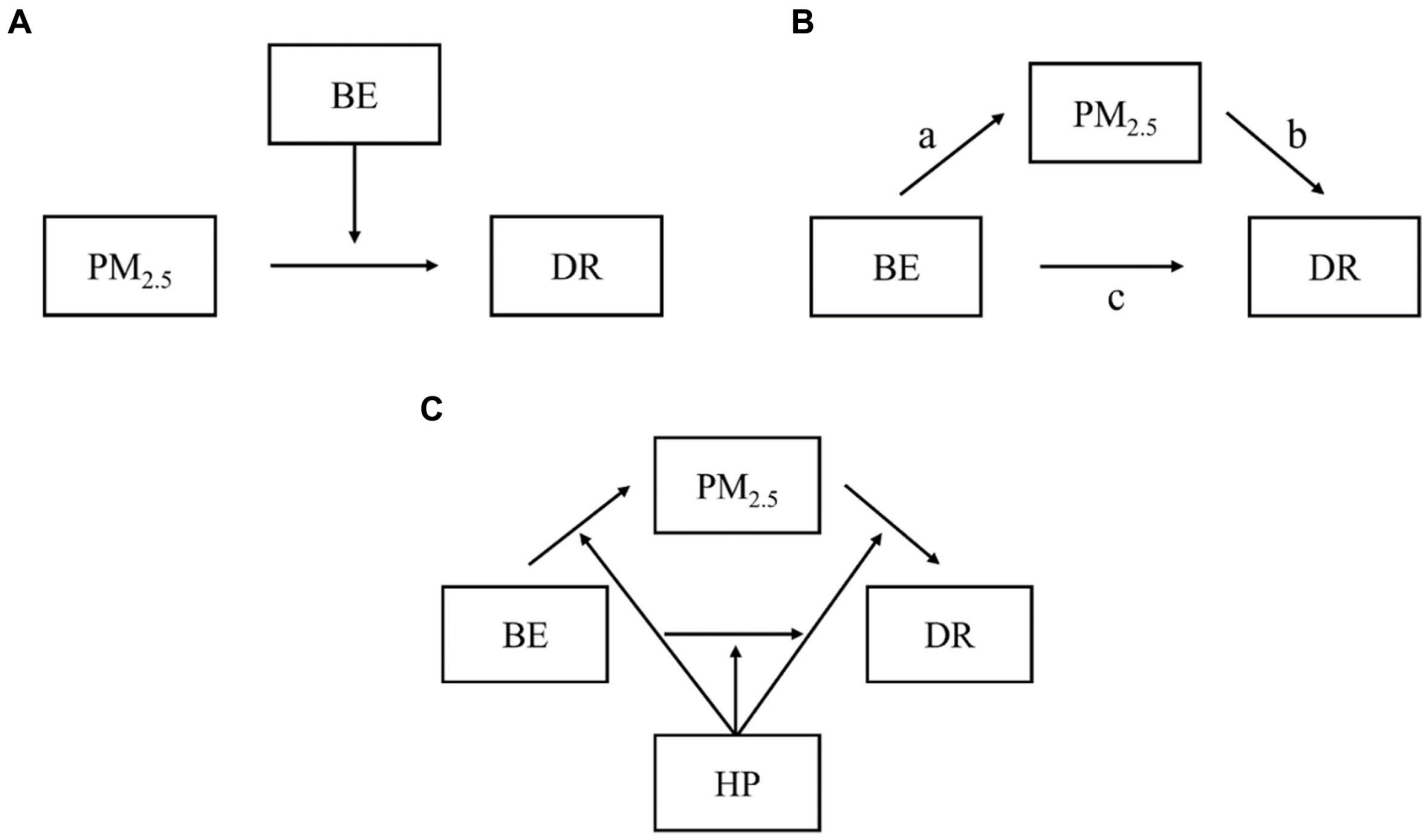
**(A)** Model 1. **(B)** Model 2. **(C)** Model 3.

Then, three models depicted in Figure 3 were run in PROCESS, an add-on for SPSS software, to explore the relationship between the four factors. Before modeling, the variables were normalized and set to zero. The purpose of Model 1 was to examine the moderating effect of built environment on PM_2.5_ and mortality rate. A significant interaction between the independent variable and the moderator confirmed the moderating effect. Model 2 was designed to investigate the mediating effect of PM_2.5_ on built environment and mortality rate. On this basis, Model 3 examined the moderating effect of housing price further.

### 2.4. Calculation of built environment

#### 2.4.1. Standardized built environment indexes

To make the data comparable and eliminate the influence of dimension, the indicators of built environment were standardized (24). Based on the results of regression model, the indicators were then divided into positive and negative indexes. The following equations were used to standardize positive and negative indexes:


$$X\prime_{ij}=\frac{X_{j}-X_{\min}}{X_{\max}-X_{\min}}$$

$$X\prime_{ij}=\frac{X_{\max}-X_{j}}{X_{\max}-X_{\min}}$$

where $X\prime_{ij}$ = standardized value; $X_{j}$ = value of indicator j; $X_{\max}$ = maximum value of indictor j; and $X_{\min}$= minimum value of indicator j.

#### 2.4.2. Weighting procedure

Indicator weights can be divided into objective and subjective weights. The entropy method utilized in this study is an objective method of weighting that is capable of reflect the utility value of the indicators. It has greater credibility and precision than the subjective weighting procedure.

The proportion of the grid index value relative to all indexes was calculated using the following equation:


$$P_{ij}=\frac{X\prime_{ij}}{\sum_{i=1}^{m}X\prime_{ij}},0\leq P_{ij}\leq 1$$

where $P_{ij}$= the proportion of the index value of grid i in the index of item j; m = the total number of grids.

The entropy was calculated using the following equation:


$$E_{j}=-k\overset{m}{\underset{i=1}{\sum }}P_{ij}\ln P_{ij}$$

$$k=\frac{1}{\ln m}$$

where $E_{j}$ = the information entropy value of index j.

The indexes were weighted using the following equations:


$$D_{j}=1-E_{j}$$

$$W_{j}=\frac{D_{j}}{\sum_{i=1}^{m}D_{j}}$$

where $D_{j}$= the information utility value of index j; $W_{j}$ = the weighting of index j.

#### 2.4.3. Value of built environment

The value of built environment was computed using the following equation. A greater built environment value indicates that the built environment is more advantageous to human health.

$$BE=\overset{n}{\underset{i=1}{\sum }}X\prime_{ij}W_{j}$$

## 3. Results

### 3.1. Pairwise correlation between built environment indicators and mortality rate

**Figure 4:**
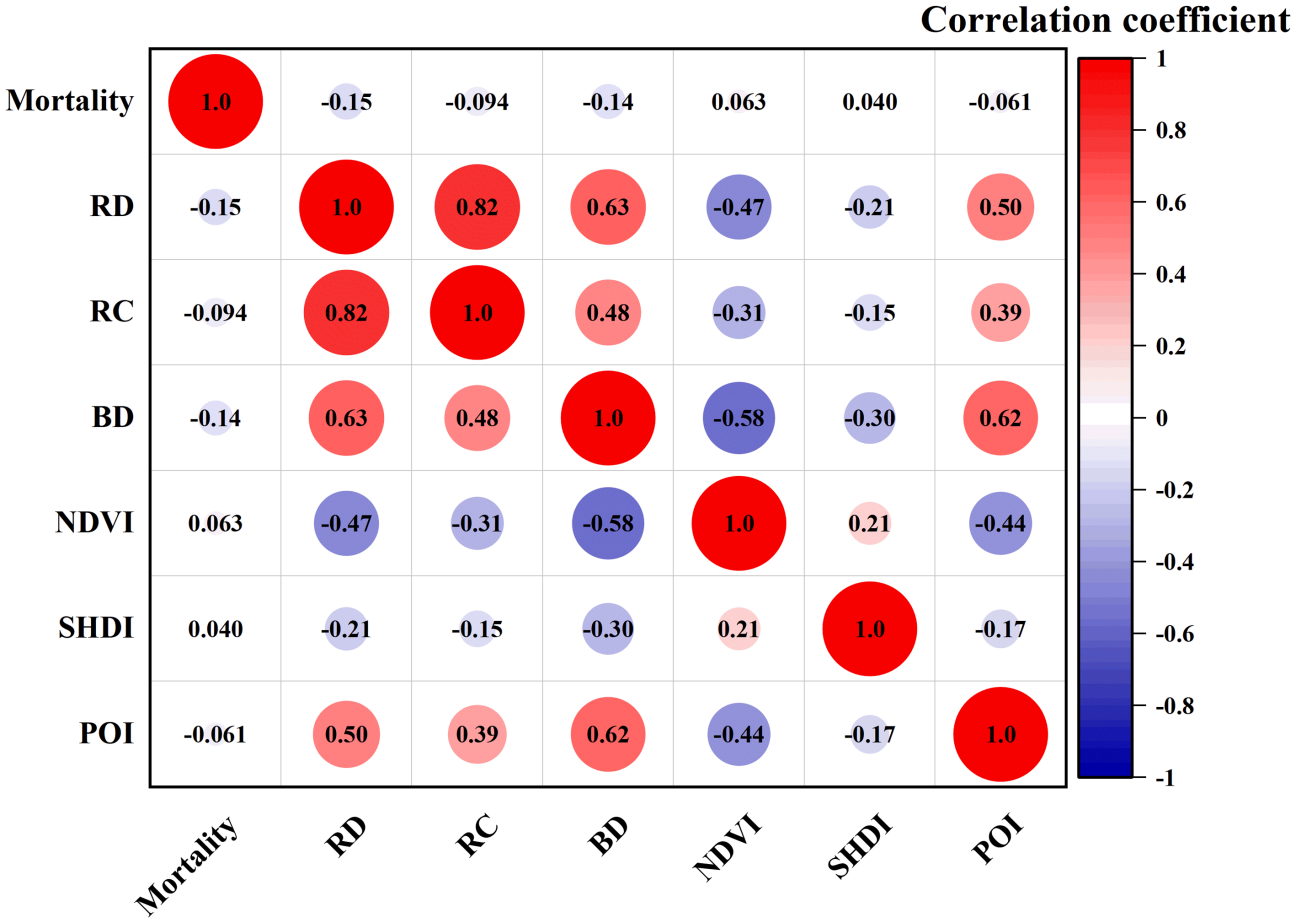
Correlation coefficient among indexes and mortality rate.

Figure 4 depicts the results for correlation analysis between built environment indicators and the mortality rate were shown in Figure 4, while Table 2 displays detailed results of regression analysis. Specific relationships between elements of built environment and health are as follows: road density, road crossing, building density, and diversity of POI all have a negative correlation with mortality rate. In contrast, NDVI and SHDI have a positive correlation with mortality rate. Based on the results of regression analysis, road density, building density and NDVI are negative indices of mortality rate, while the road crossing and diversity of POI are positive indices.

**Table 2:** Results of regression models.

Independent variable	*B*	S.E.	*β*	*P*
Road density	−0.081	0.017	−0.184	0.000
Road crossing	0.085	0.035	0.079	0.017
Building density	−0.034	0.008	−0.120	0.000
NDVI	−0.015	0.008	−0.043	0.068
Diversity of POI	5.175	2.308	0.055	0.025
SHDI	−0.001	0.006	−0.004	0.826
PM_2.5_	0.005	0.001	0.128	0.000
BE	−0.060	0.010	−0.189	0.000
HP	0.023	0.000	−0.110	0.000

B is the unstandardized coefficient; S.E. is the standard error; β is the standardized coefficient.

### 3.2. Pairwise correlation between air pollution, built environment, housing price, and mortality rate

**Figure 5:**
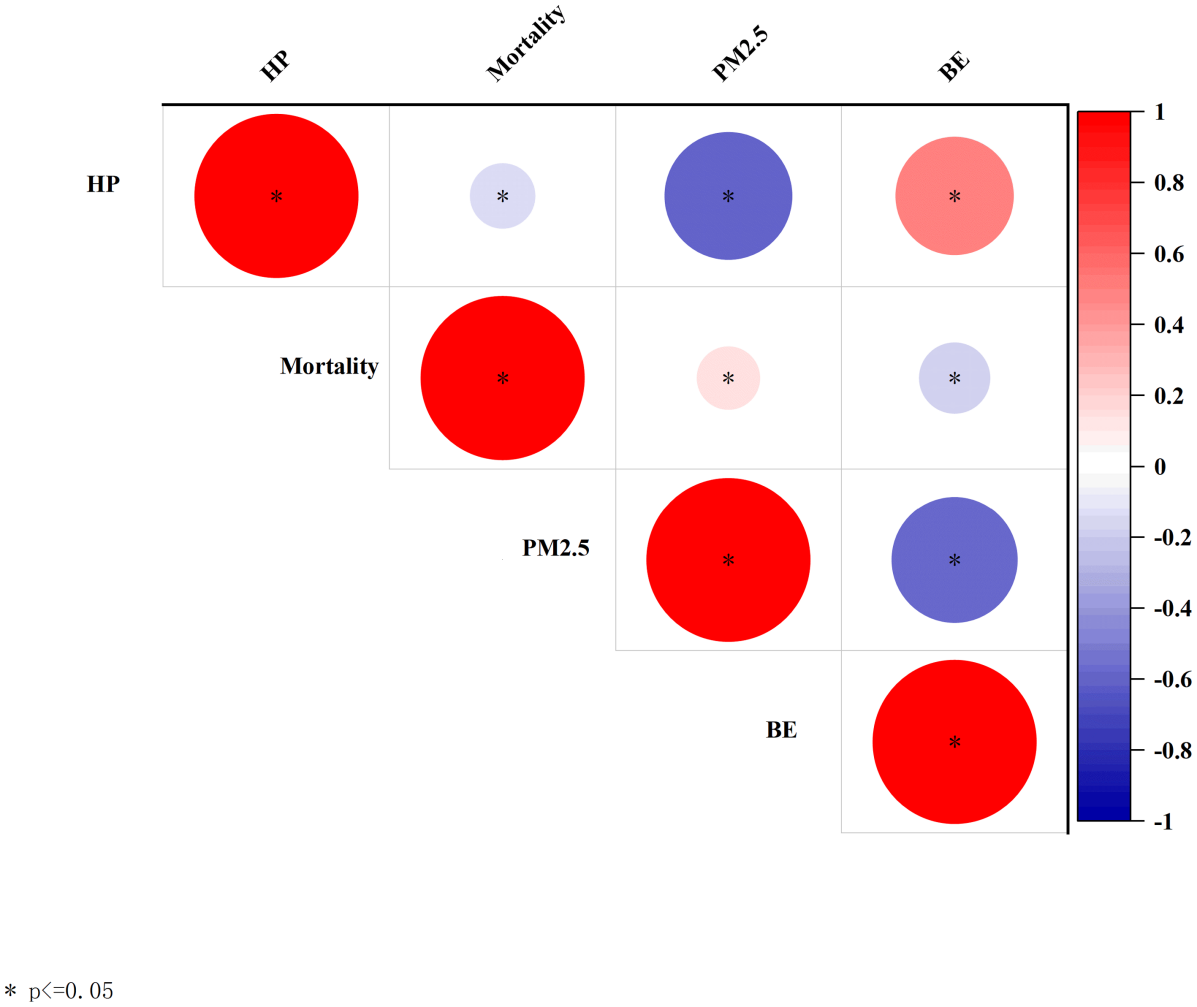
Correlation coefficient among HP, BE, PM_2.5_, and mortality rate.

The results for correlation analysis were shown in Figure 5, and the regression analysis results were presented in Table 2. There were statistically positive associations between PM_2.5_ and mortality rate. Built environment and housing price exhibited negative and significant correlations with mortality rate and PM_2.5_.

### 3.3. Spatial patterns of air pollution, built environment, housing price, and mortality rate

**Figure 6:**
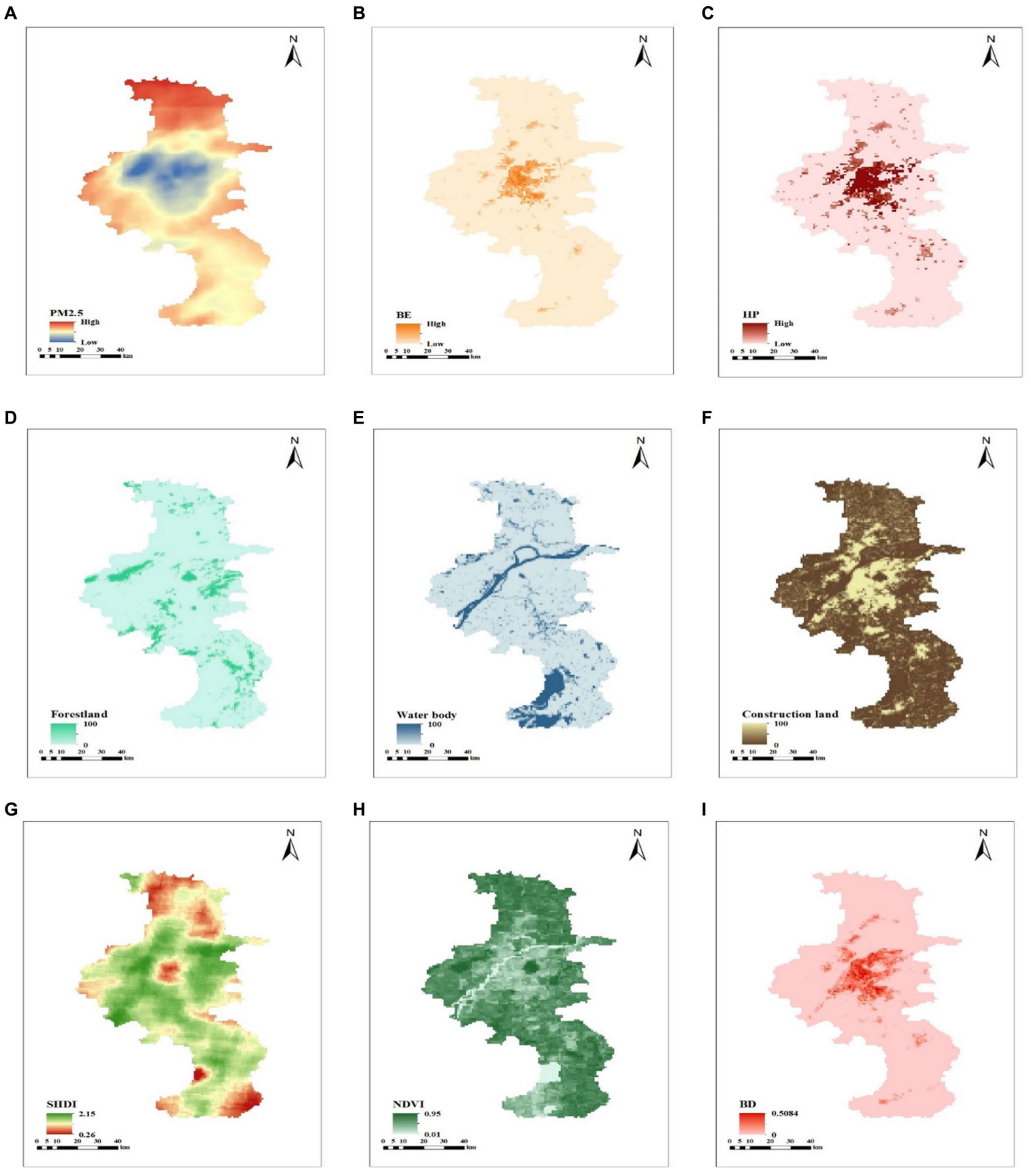

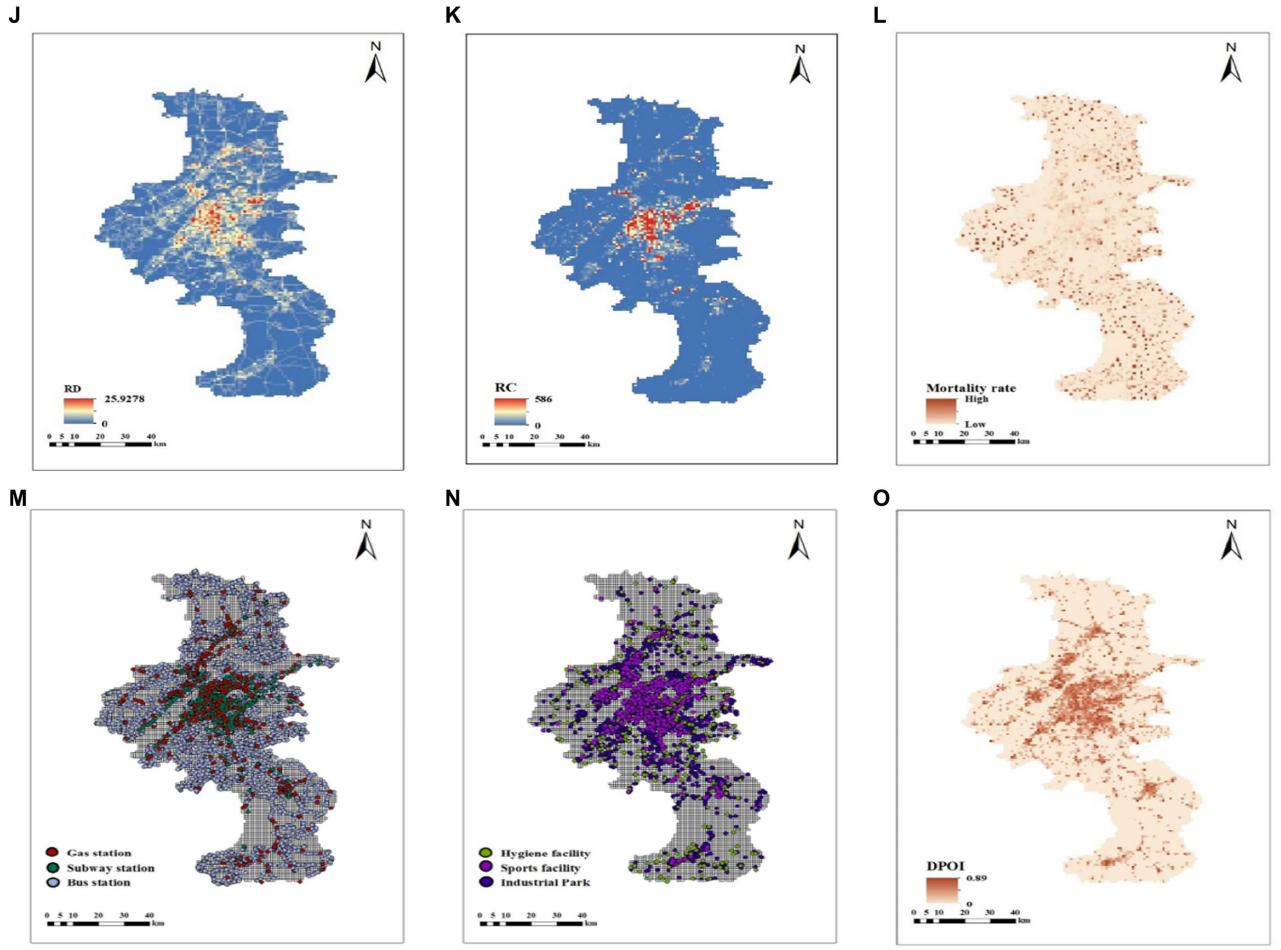
Spatial patterns of the built environment. **(A)** Concentration of PM_2.5_. **(B)** Value of built environment. **(C)** Housing price. **(D)** Proportion of forestland. **(E)** Proportion of water body. **(F)** Proportion of construction land. **(G)** SHDI. **(H)** NDVI. **(I)** Building density. **(J)** Road density. **(K)** Road count. **(L)** Mortality rate. **(M)** POI of infrastructures. **(N)** POI of infrastructures. **(O)** Diversity of POI.

Figure 6 depicts the spatial patterns of air pollution, built environment elements, housing price and mortality rate. All of them are discovered to have a high level of agglomeration. The central region is more urbanized, with dense structures, roads, and an abundance of public service facilities, but the NDVI is lower. In close proximity to the city center, the total level of built environment and housing price are higher while the mortality rate is lower. The center of the study area had comparatively low PM_2.5_ concentrations whereas the southeast and northeast of the urban districts were more heavily polluted by PM_2.5_, consistent with previous research (25).

This study first quantified the value of built environment of each grid. Then, with the aid of the ArcGIS visualization function, it is determined that majority of grids with a high built environment value are concentrated in the center of Nanjing. In contrast, grids with a low built environment value are located on the outskirts of Nanjing. On basis of this result and the spatial distribution of PM_2.5_ concentration, housing price, and mortality rate, it is also demonstrated that the urban core has a high PM_2.5_ concentration, a high housing price and a low mortality rate. In contrast, the urban suburb has a low PM_2.5_ concentration, a low housing price and a high mortality rate. The grids in the urban center and urban suburbs have revealed glaring disparities in built environment and health. Consequently, the city center and city suburb arrangements that satisfy the before mentioned conditions are chosen. Ultimately, city center grids and city suburb grids were identified as two categories that require comparison and discussion in order to explore whether built environment inequalities exacerbate health inequalities.

### 3.4. Relationships between built environment, air pollution, and mortality rate: moderating model

Model 1 (Figure 3A) was used to test the moderating effect of built environment on the primary relationship between PM_2.5_ and mortality rate. As an indicator of the moderating effect between PM_2.5_ and mortality rate, the coefficient of the interaction item “built environment × air pollution” was employed. The interaction term between built environment and PM_2.5_(BE*PM_2.5_) had a negative and statistically significant effect on mortality rate implementation in the city center (*b* = −0.2824, *p* = 0.0001). Similarly, the impact of the interaction term (BE*PM_2.5_) was significantly negative in urban suburbs (*b* = −0.9464, *p* = 0.0000). In suburban areas, both the adjustment coefficient and significance level were greater than in urban areas.

Table 3 demonstrates the results. It shows that built environment can negatively moderate the relationship between PM_2.5_ and mortality in both city center and the suburbs, indicating that a high level of built environment can help mitigate the negative health effects of PM_2.5_. Compared to the city center, the suburban built environment has a greater moderating effect.

**Table 3:** Results of moderating effects of built environment.

Independent variable		*β*	SE	*P*	LLCI	ULCI
City center	PM_2.5_	0.1477	0.0339	0.0000	0.0808	0.2145
	BE	0.0012	0.0199	0.9504	−0.038	0.0405
	PM_2.5_*BE	−0.2824	0.0712	0.0001	−0.4228	−0.1421
City suburb	PM_2.5_	0.1192	0.0284	0.0000	0.0633	0.1751
	BE	−0.031	0.1178	0.7929	−0.2629	0.2010
	PM_2.5_*BE	−0.9464	0.2116	0.0000	−1.363	−0.5299

### 3.5. Associations between built environment, air pollution, and mortality rate: a model of mediation

Model 2 (Figure 3B) examines the mediation effect of PM_2.5_ on the previously discussed associations between built environment and mortality rate discussed above. Table 4 shows the results of the mediation analyses of city center. Path a revealed a negative relationship between built environment and PM_2.5_ in city center, (*p* = 0.0274, *b* = −0.2143). PM_2.5_ was positively associated with mortality rate in relation to path *b* (*p* = 0.0029, *b* = 0.0144). Path c revealed a negative relationship between built environment and mortality rate (*p* = 0.0000, *b* = −0.0732). The coefficient of mediation effect is-0.0031 according to the Bootstrap test of deviation correction (95%CI: −0.0086, −0.0001).

**Table 4:** Results of mediating effects of PM_2.5_.

Independent variable		*β*	SE	*t*	*P*	LLCI	ULCI
City center	Total effect	−0.0763	0.007	−10.9694	0.0000	−0.09	−0.0626
	Direct effect	−0.0732	0.0069	−10.5975	0.0000	−0.0868	−0.596
	Indirect effect	−0.0031	0.0023			−0.009	−0.0001
City suburb	Total effect	−0.213	0.0458	−4.6504	0.0000	−0.3031	−0.1228
	Direct effect	−0.1527	0.048	−3.1795	0.0016	−0.2473	−0.0582
	Indirect effect	−0.0602	0.0178			−0.0993	−0.0295

Path a revealed a negative relationship between built environment and PM_2.5_ in urban suburbs (*p* = 0.0000, *b* = −0.14863). PM_2.5_ was positively associated with mortality rate along path b (*p* = 0.0005, *b* = 0.0405). Path c revealed a negative relationship between built environment and mortality rate (*p* = 0.0016, *b* = −0.1527). The coefficient of mediation effect is −0.0602 according to the Bootstrap test of deviation correction (95%CI: −0.098, −0.0308). Table 4 shows the results of the mediation analyses of city center and suburb.

Overall, the results indicate that PM_2.5_ mediated the relationship between built environment and mortality rate in both city center and city suburb. In addition to having direct effects on health, the built environment indirectly affects pollutant concentrations and, by extension, thus health.

### 3.6. Associations between built environment, air pollution, housing price, and mortality: a model of mediated moderation

Model 3 was used (Figure 3C) and the results are shown in Table 5. In the city center, there was a significant interaction between built environment and housing price (*b* = 1.1474, *Δ*$R^{2}=$ 0.0197, *p* = 0.0232) and a negative relationship between built environment and PM_2.5_ meaning that as built environment scores increased,PM_2.5_ scores decreased. This linear relationship was observed when housing price were one standard deviation (SD) below the average; *b* = −0.2171, 95%CI [−0.4323, −0.0019], *p* = 0.048 < 0.05,when housing price were average; *b* = −0.0912, 95%CI [−0.2708, 0.0884], *p* = 0.3179 > 0.1, and when housing price one SD above the average; *b* = 0.1471, 95%CI [−0.1178, 0.4121], *p* = 0.2749 > 0.1. This suggested that, compared to high housing price in the city center, low housing price amplified the effect of built environment on PM_2.5_.

**Table 5:** Results of mediated moderating effects of housing price.

	Path	HP	*β*	SE	*P*	BootLLCI	BootULCI
City center	BE→PM_2.5_ (direct effect)	M-1SD	−0.2171	0.1092	0.00480	−0.4323	−0.0019
		M	−0.0912	0.0911	0.3179	−0.2708	0.0884
		M + 1SD	0.1471	0.1344	0.2749	−0.1178	0.4121
	BE→DR (direct effect)	M-1SD	−0.2853	0.0734	0.0001	−0.4298	−0.1407
		M	−0.1678	0.0516	0.0013	−0.2696	−0.0661
		M + 1SD	−0.0504	0.0718	0.4839	−0.1919	0.0911
	PM_2.5_ → DR (direct effect)	M-1SD	0.0544	0.0169	0.0015	0.0211	0.0877
		M	0.0318	0.0151	0.036	0.0021	0.0614
		M + 1SD	0.0091	0.0214	0.6709	−0.0331	0.0513
	BE→PM_2.5_ → DR (indirect effect)	M-1SD	−0.0282	0.0158		−0.0656	−0.0043
		M	−0.0166	0.0108		−0.0407	0.0019
		M + 1SD	0.0034	0.0096		−0.014	0.0249
City suburb	BE→DR (direct effect)	M-1SD	−0.2853	0.0734	0.0001	−0.4298	−0.1407
		M	−0.1678	0.0516	0.0013	−0.2696	−0.0661
		M + 1SD	−0.0504	0.0718	0.4839	−0.1919	0.0911
	PM_2.5_ → DR (direct effect)	M-1SD	0.0465	0.0169	0.0064	0.0132	0.0797
		M	0.0216	0.0153	0.1594	−0.0085	0.0517
		M + 1SD	−0.0034	0.0216	0.8764	−0.0459	0.0391

In addition, the interaction term between PM_2.5_ and housing price has a negative and significant impact (*b* = −0.0608, *p* = 0.0648 < 0.1, $\Delta R^{2}=$0.0092). The correlation between PM_2.5_ and mortality rate indicated that as PM_2.5_ levels increased, so did mortality rates. This linear relationship was observed when housing price were one SD below the average; *b* = 0.0544, 95%*CI* [0.0211, 0.0877], *p* = 0.0015 < 0.01, when housing price were average; *b* = 0.0318, 95%CI [0.0021, 0.0614], *p* = 0.036 < 0.1, and when housing price one SD above the average; *b* = 0.0091, 95%, *CI* [−0.0331, 0.0513], *p* = 0.6709 > 0.1. It appeared that, in comparison to high housing price in the city center, low housing price mitigated the effect of PM_2.5_ on mortality rate.

Housing price can also moderate the relationship between built environment and mortality (*b* = 0.1425, *p* = 0.0002 < 0.001, *Δ*$R^{2}=$ 0.0374). Built environment has a negative correlation with the mortality rate, so as built environment increased, mortality rate decreased. This linear relationship was observed when housing price were one SD below the average; *b* = −0.2853, 95%CI [−0.4298, −0.1407], *p* = 0.0001, when housing price was average; *b* = −0.1678, 95%CI [−0.2696, −0.0661], *p* = 0.0013, and when housing price one SD above the average; *b* = −0.0504, 95%, CI [−0.1919,0.0911], *p* = 0.4839 > 0.1. In comparison to high housing price in city center, low housing price amplified the effect of built environment on mortality.

The interaction between built environment and housing price is insignificant in city suburbs (*p* = 0.7832 < 0.1),while interaction between PM_2.5_ and mortality rate is significant (*b* = −0.1856, *p* = 0.0375 < 0.1, $\Delta R^{2}=$0.0155). Moreover, housing price can moderate the relationship between built environment and mortality rate (*b* = 0.8749, *p* = 0.0222, $\Delta R^{2}=$0.0189). The negative association between built environment and mortality rate was stronger in areas with low housing price (1 SD below mean value), than that among places with high housing prices (1 SD above mean value). Housing price mitigates the negative health effects of t PM_2.5_ and amplify the positive health effects of built environment. Overall, the effect of housing price moderation in city center is more considerable than in city suburbs.

## 4. Discussion

### 4.1. Built environment moderates the health effects of PM_2.5_

First, a spatial distribution analysis reveals that the city center has a high level of built environment. Conversely, the built environment of the urban suburb is deficient. It is important to note that urban center and urban suburb are two typical areas of the residential segregation phenomenon in Nanjing. Some international literatures have focused on the poverty-inequality-environment relationship (26). The findings of built environment in Nanjing are consistent with those of previous international studies conducted in Latin American (27), which demonstrated that segregation within cities is accompanied by an unequal distribution of environmental characteristics and significant environmental differences between social classes. Specifically, there is a direct positive correlation between “urban green space” and “urban spatial isolation” factors. In addition, both the PM_2.5_ concentration and mortality rate in Nanjing were found to be significantly polarized by this study. In particular, the PM_2.5_ concentration and mortality rate are relatively low in city center, while the PM_2.5_ concentration and mortality rate are relatively elevated in city suburb. The is the same result of studies in south-central Chile (28), where socioeconomic heterogeneity in ambient particulate matter concentrations was found. A study in Oslo, Norway (29), also proved that impoverished citizens are surrounded by less blue-green space and are more vulnerable to air pollution. The unequal spatial distribution of built environment, air pollution and health is a global problem.

The relationship between built environment, mortality rate and PM_2.5_ are further investigated based on this phenomenon. It is important to note that a high level of built environment can help mitigate the negative impact of PM_2.5_ on human health, confirming the effect of built environment on health disparities. Surprisingly, it is discovered that built environment has a greater moderating effect in city suburbs than in the city centers. This may be due to the fact that vulnerable groups, such as migrants at lower income, reside primarily in China’s urban peripheries that have weak social and built environments (30). Poor and remote communities are typically exposed to higher levels of air pollution and face greater health risks as a result (31). Another possible explanation is the uneven distribution of public hospitals and other medical resources in China. In city suburbs, communities with an abundance of medical resources have fewer health risks than other communities.

### 4.2. Mediating role of PM_2.5_ in built environment and health

This study considered the comprehensive PM_2.5_ concentration in Nanjing, including stationary sources and mobile sources. Results showed that PM_2.5_ mediates the relationship between built environment and mortality rate in both city center and city suburb. According to the spatial distribution of built environment elements in Nanjing, it may be due to the correlation between transportation-related elements of the built environment and PM_2.5_ concentrations. Traffic volume on roads (32, 33), road width, bus route length, and the number of bus stops (34, 35) have been examined as determinants of PM_2.5_ in previous studies. As is shown in Figure 5, there are numerous intersections and subway stations in the center of Nanjing, and many bus stations are concentrated in the suburbs. Traffic can be an important source of pollution in Nanjing. Contrarily, greenness, lakes and wetlands are mainly located in suburb of Nanjing, where air pollutants are deposited and absorbed (36). Greener built environment located in the suburbs has the advantage of mitigating PM_2.5_.

### 4.3. Housing prices influence health inequalities within cities

In China, housing price is a motivator for residential segregation. Rapidly rising housing prices and increasing housing inequality reshape the Chinese urban landscape and have a negative impact on the well-being of city dwellers (37, 38). Thus, housing prices in relation to the built environment is considered in order to determine if housing prices can moderate the effect of built environment on health, which may exacerbate health inequalities within cities. It is discovered that housing price in Nanjing decreases from the city center to the outskirts. The results also indicate that housing price can effectively moderate the relationship between built environment and mortality rate, which is mediated by air pollution in city center. Specifically, housing price amplified the positive effect of built environment on PM_2.5_ and mortality rate, while attenuating the negative effect of PM_2.5_ on mortality rate, which is especially pronounced in relatively low-price areas of city center. In suburban areas, the effect of PM_2.5_ on health is mitigated by housing price, while the effect of built environment on health is enhanced.

It may be because residents with higher income prefer to invest in private greening (39). The magnitude of the moderating effect of built environment is determined by people’s income and capacity to pay. Increasing numbers of people today have a heightened awareness of green ecology and pay greater attention to the air quality near their homes. For example, a study discovered that residences within two meters of a green zone could have higher housing prices, with a prospective increase of nearly 25 billion yuan in Beijing (40). Therefore, housing prices and built environment interact and ultimately influence health. Moreover, housing prices reflect the importance placed on air pollution (38). For the sake of quality of life and physical health, it is evident that residents are willing to pay higher housing prices to reside in areas with excellent air quality as opposed to areas with high air pollution (41). This explains the moderating effect of housing prices on the relationship between PM_2.5_ and mortality rate.

## 5. Conclusion

This study examined the associations between built environment, PM_2.5_, housing price and mortality rate in Nanjing, China, to cast light on health disparities under residential segregation. Beginning with a moderating analysis, the effect of built environment on PM_2.5_ and mortality rate were examined. Built environment is then incorporated into the mediation effect model to determine if the built environment can affect health by influencing PM_2.5_. Finally, a moderated mediating effect model was used to examine whether housing prices moderate the relationship between built environment, air pollution, and health.

The empirical findings presented here indicate: first, the built environment can reduce the negative response of the mortality rate to PM_2.5_. However, the moderating effect of built environment differs between urban center and urban suburb. The positive effect of built environment on health is greater in city suburb than in city center. Second, no matter in urban centers or urban suburbs, the built environment can also indirectly influence PM_2.5_ concentrations and thus health. Third, housing price will mitigate the negative health effects of PM_2.5_ and strengthen the positive health effect of built environment. Overall, the moderation effect of housing price is greater in city center than in city suburbs, which may exacerbate health inequalities.

### 5.1. Policy and suggestions

Based on the moderating analysis, the mediating analysis and the moderated mediating analysis, a close relationship between health, built environment, air pollution and housing price is discovered in Nanjing city, which ultimately results in unequal spatial aggregation. The following mortality risk mitigation measures are recommended from the perspective of built environment: to avoid the “environment-health-poverty” trap (42) to the greatest extent possible, more public resources should be invested in the construction of urban suburbs, and the balanced development of infrastructure, especially medical facilities, transportation facilities, and athletic facilities should receive special attention. To mitigate the mortality risk from air pollution, the development of the city center should not disregard the degree of land use blending (43) and should focus on enhancing the diversity of land cover, particularly water area and greenness (44). In addition, increasing the number of urban parks in center areas can directly affect urban temperature (45), thereby enhancing the thermal comfort of residents and promoting physical activity.

### 5.2. Strengths and limitations

The study concludes that residential segregation is accompanied by unequal distribution of built environment, with the effect of PM_2.5_ and housing price exacerbating health disparities. It contributes to research fields about segregation and welfare. Furthermore, this study quantifies the built environment, mortality rate, housing price, and air pollution at the grid scale. The 1 km × 1 km grid scale is more conducive to characterizing and disclosing the influence mechanism of the four factors than previous regional-perspective studies.

However, this study has a few limitations. Firstly, it revealed the correlations between four factors only in the case of Nanjing. More regions will be considered to examine further the importance of building environment and housing price to air pollution and human health in future research. In addition, this study has confirmed the moderating effects of built environment and housing prices on air pollution and health, but some other factors, such as climate change (19, 46), that can also impact health were neglected. Numerous non-communicable diseases would be promoted and exacerbated by climate change’s increased environmental exposures (47). Consequently, climate change can be the focus of future research.

## Data availability statement

The original contributions presented in the study are included in the article/supplementary material, further inquiries can be directed to the corresponding author.

## Author contributions

PW and YD: conceptualization. YD: methodology, formal analysis, and data curation. CW: software. YD and CW: validation. JW and YD: investigation. LH: resources. YD, CW, and JW: writing—original draft preparation. CW and JW: writing—review and editing. JW: visualization. PW: supervision, project administration, and funding acquisition. All authors have read and agreed to the published version of the manuscript.

## Funding

This work was supported by the National Natural Science Foundation of China [Grant No. 51908249], the Natural Science Foundation of Jiangsu Province [Grant No. SBK2023022191], the Natural Science Foundation of the Jiangsu Higher Education Institutions of China [Grant No. 19KIB560012], the High-level Scientific Research Foundation for the introduction of talent for Jiangsu University [Grant No. 18JDG038], the Innovative Approaches Special Project of the Ministry of Science and Technology of China [Grant No. 2020IM020300], and the Science and Technology Planning Project of Suzhou [Grant No. ST202218].

## Conflict of interest

The authors declare that the research was conducted in the absence of any commercial or financial relationships that could be construed as a potential conflict of interest.

## Publisher’s note

All claims expressed in this article are solely those of the authors and do not necessarily represent those of their affiliated organizations, or those of the publisher, the editors and the reviewers. Any product that may be evaluated in this article, or claim that may be made by its manufacturer, is not guaranteed or endorsed by the publisher.

## Footnotes

^1^ http://www.bigemap.com/

^2^ https://nj.lianjia.com

^3^ https://icd.who.int/browse10/2010/en#/IX
